# From Research to Policy: The WHO Experience With Developing Guidelines on the Potential Risk of HIV Acquisition and Progestogen-Only Contraception Use

**DOI:** 10.9745/GHSP-D-17-00278

**Published:** 2017-12-28

**Authors:** Leo Han, Eva Patil, Nancy Kidula, Mary Lyn Gaffield, Petrus S. Steyn

**Affiliations:** a Department of Obstetrics and Gynecology, Oregon Health & Science University, Portland, OR, USA.; b WHO AFRO, Intercounty Support Team for Eastern and Southern Africa, Harare, Zimbabwe.; c Department of Reproductive Health and Research, World Health Organization, Geneva, Switzerland.

## Abstract

To develop guidance for women at high risk of HIV, WHO carefully considered the risks of maternal morbidity and mortality from unintended pregnancy against possible increased risk of HIV acquisition with injectable use. Among the many challenges: (1) balancing timeliness of changing the guidance against the potential impact of it; (2) engaging a range of stakeholders; (3) translating complex research and policy messages to clients; (4) needing additional research; and (5) monitoring and evaluating successes and challenges with implementing new guidelines.

The complex relationship between research and global health policy is no better illustrated than by the ongoing discussion regarding the association between HIV acquisition and hormonal contraception, and in particular, progestogen-only injectable contraceptives (POIs). Despite an array of epidemiological, translational, and basic science research, the question persists as to whether there exists a causal increased risk of HIV acquisition in women who use POIs. Most recently, in August 2016 Polis et al. published in the journal *AIDS* an updated systematic review of the available clinical literature.^1^ The authors concluded that the highest-quality studies suggest a hazard ratio of 1.4 (95% confidence interval, 1.2 to 1.7) for HIV acquisition in women who use the POI depot medroxyprogesterone acetate (DMPA).

While the currently available scientific evidence demonstrates substantial uncertainty as to whether or not the association between DMPA use and HIV acquisition is causal, the need for up-to-date policy reflecting current findings is often more urgent than waiting for definitive research. The implications of this research for women in areas with high HIV prevalence, such as sub-Saharan Africa, are significant as many of the same countries with high HIV prevalence also experience high maternal morbidity and mortality. Contraceptive use plays a critical role in preventing maternal morbidity and mortality by helping women avoid unintended pregnancy.^2^ But unmet need for contraception in sub-Saharan Africa (21%) is the highest in the world.^3^ Furthermore, many countries in sub-Saharan Africa often have a limited variety of available contraceptive methods, and POIs such as DMPA and norethisterone enanthate (NET-EN) are familiar and widely used methods—indeed, DMPA is the single most widely used method in most sub-Saharan African countries (Figure 1).^5^ For example, over 46% of modern method contraceptive users in the Southern Africa region use POIs.^3^^,^^5^ At the country level, POIs comprise 56% of modern method use in Uganda, 51% in Ethiopia, and 46% in Kenya.^6^

**FIGURE 1.**
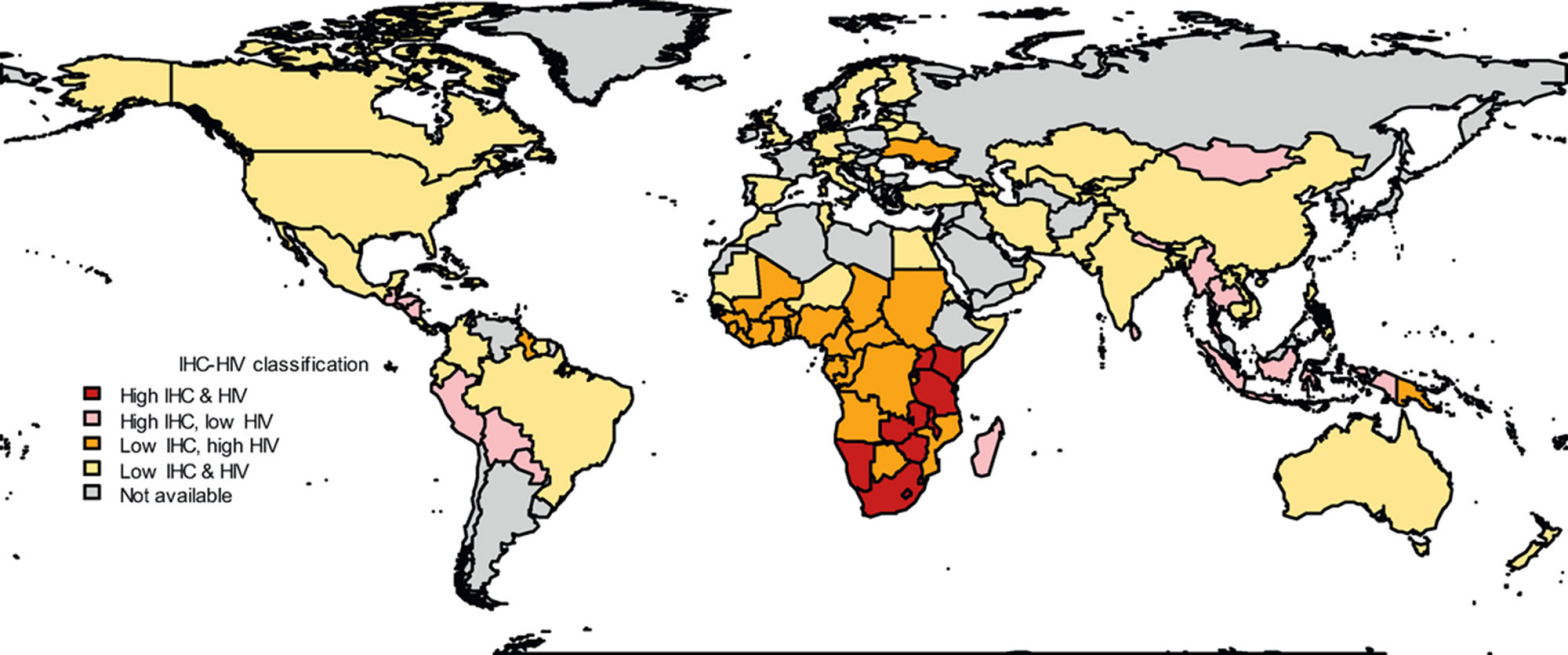
Prevalence of Injectable Contraceptive Use and HIV Prevalence by Country Abbreviation: IHC, injectable hormonal contraception. Note: sub-Saharan African countries in red have both high HIV prevalence and high injectable hormonal contraception use. Source: Reproduced from Butler et al. 2013^4^ with permission.

Removing POIs from the contraceptive method mix in countries with high HIV prevalence as a reflexive response to uncertain associations could result in a large decrease in contraceptive use, which could in turn result in a marked increase in maternal morbidity and mortality from unintended pregnancy. These complicated risk-benefit scenarios have been modeled and indeed demonstrate that removing POIs without the majority of women switching to an alternative highly effective modern contraceptive method would result in more maternal deaths than HIV cases averted,^7^^,^^8^ and an estimated 9,000 life-years would be lost per 100,000 women.^8^ The alternative methods, such as intrauterine devices (IUDs) and contraceptive implants, are far less popular with women in areas of high HIV prevalence such as sub-Saharan Africa, and the number of women needed to switch to these methods in order to reach net neutral mortality is unrealistic in countries with the highest mortality rates.^5^^,^^9^ For these reasons, conclusions from research like the recent systematic review must be carefully interpreted and communicated to key stakeholders and thoughtfully translated into appropriate practice.

Conclusions from research on the potential increased risk of HIV acquisition with use of injectable contraceptives must be carefully interpreted and thoughtfully translated into appropriate practice.

The World Health Organization (WHO), in an effort to provide evidence-based sexual and reproductive health guidance to its member states, continually reviews current research and creates and disseminates recommendations to reflect the best health practices with a human rights-based approach.^10^ Our experience with the implementation of these evidence-based guidelines reflects how challenging creating health care policy can be. Policy must not only keep up with a changing research landscape but also account for the needs and concerns of multiple stakeholders as well as the people it ultimately will affect. We highlight several aspects of this experience to show just how challenging this process can be.

## EVOLUTION OF WHO INJECTABLE USE POLICY

The cornerstone of WHO guidance on contraceptive safety is the maintenance of an up-to-date reference for policy makers, program managers, and health care providers called the *Medical Eligibility Criteria for Contraceptive Use* (MEC).^11^ The MEC, now in its fifth edition, contains more than 2,000 recommendations for 25 different contraceptive methods and addresses more than 80 different medical conditions or patient characteristics. It uses a four-tiered classification level stratified by safety for using a contraceptive method given a specific condition (Table). In general, for situations where clinical judgment is limited (for example, in the case of frontline health workers who are often the main POI providers), a woman with a category 1 or category 2 condition can generally use the method, whereas a woman with a category 3 or category 4 designation should not.

**TABLE.** Four-Tiered Categorization of Contraceptive Method Eligibility in the World Health Organization's *Medical Eligibility Criteria for Contraceptive Use*^11^

Category	Description
1	A condition for which there is no restriction for the use of the contraceptive method
2	A condition where the advantages of using the method generally outweigh the theoretical or proven risks
3	A condition where the theoretical or proven risks usually outweigh the advantages of using the method
4	A condition which represents an unacceptable health risk if the contraceptive method is used

**FIGURE 2.**
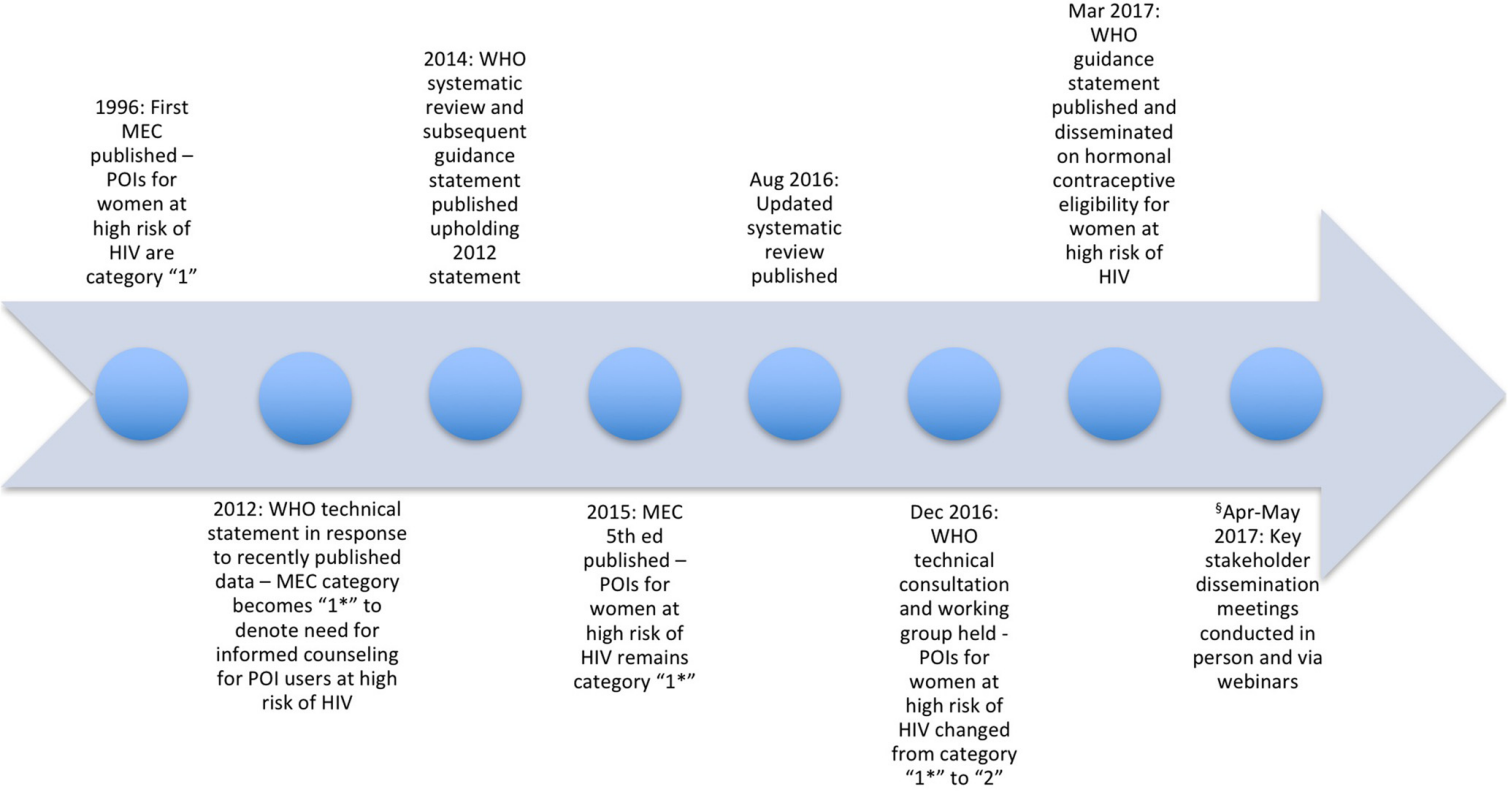
WHO Timeline of Events From Publication of Research on Possible Increased Risk of HIV Acquisition in POI Users to Guideline Dissemination to Policy Implementation Abbreviations: MEC, *Medical Eligibility Criteria for Contraceptive Use*; POI, progestogen-only injectables; WHO, World Health Organization. § Two webinars in February 2017 prepared 75 WHO country office team members, ministry of health representatives, family planning donors, and researchers for the publication of the updated guidelines. Following publication, a key stakeholder dissemination meeting was held in Johannesburg, South Africa, in April 2017 with 59 participants. Additional webinars to further disseminate the new guidance were held in April 2017 (156 participants) and May 2017 (98 French-speaking participants).

The MEC category for POI contraceptive use in women who are at high individual risk for HIV acquisition started as category 1 in the first MEC and stayed a “1” through the fourth edition in 2009 (Figure 2). Up until that time, trials primarily consisted of smaller observational studies with mixed findings, many in populations of female sex workers, which limited generalizability.^12^ Newer literature with positive associative findings, including a large analysis of serodiscordant couples from Heffron et al.,^13^^,^^14^ led to an issuance of a WHO technical statement in 2012 addressing the issue and recommending that category 1 be retained. However, WHO added a specific clarification at that time (denoted by a “1*”) that noted the findings of the potential increased risk of HIV acquisition with POI use in women who are at higher risk of acquiring HIV. WHO also subsequently sponsored a systematic review to synthesize and evaluate the existing literature.^12^ In March 2014, a guideline development group reviewed and reaffirmed the “1*” status and included the upheld recommendation in the current fifth edition of the MEC.

As new data were published, WHO updated the systematic review on the association between hormonal method use and risk of HIV acquisition, resulting in a 2016 publication.^1^ These new data, along with a technical consultation with experts in the field in December 2016, led to the most recent change in the MEC for use of POIs by women at high individual risk for HIV, from category 1* (no restriction) to category 2 (benefits outweigh risks). The rationale for this change was that the category “1*” designation had not resulted in the intended increased counseling around potential use of POI methods by high-risk individuals, which was explained within the added clarification. The official WHO updated guidance statement was published on March 2, 2017.^15^

## CHALLENGES OF GUIDELINE DEVELOPMENT

### Timeliness

We recognize policy implementation often lags significantly behind research publication. This is usually the result of due diligence to investigate the potential impact of policy changes, including unintended consequences. In considering the possibility of changing the MEC recommendation for POIs, WHO consulted experts in infectious diseases, obstetrics and gynecology, evidence-based medicine, epidemiology, and pharmacology; stakeholders representing key populations at high risk of HIV infection; and managers of public health programs in highly affected settings to assess the strength of the evidence and establish consensus around global messaging prior any guideline changes. Given the high-stake implications of this topic—and the potential for the many nuances to be both oversimplified and sensationalized—WHO deemed a delay in addressing the issue until the next revision of the entire MEC (which takes place approximately every 5 years) was not an option. While the faster response shortened the interval from research to policy, it also meant that stakeholders and health care providers were asked to deal with more frequent changes to guidelines and adjust accordingly. WHO recognized that for some countries and organizations, this called for additional resources that were already limited.

### Engagement

**FIGURE 3.**
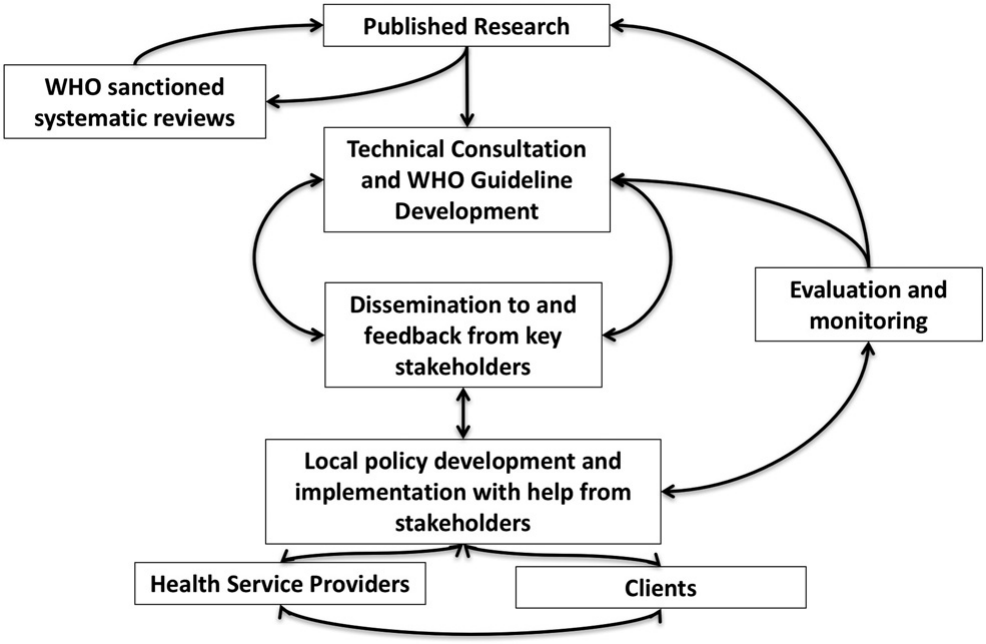
WHO Process of Translating Research to Health Policy Abbreviations: WHO, World Health Organization. The WHO reviews newly published research that has the potential to impact health policy during technical consultations with experts in the field. WHO may commission a systematic review of the topic to help collate data and interpret the potential global public health impact of the findings. Experts at the technical consultation will come to consensus on how the research should inform WHO guidelines. The guidelines are then disseminated to key stakeholders (e.g., ministries of health, NGOs, donors, and civil society) for review and comments. Stakeholders help develop policies at the national to the local service delivery levels and communicate updates to service providers and clients. Service providers and clients may provide feedback about policies, resulting in further changes. WHO and stakeholders evaluate and monitor the policies and their implementation, which then informs guideline updates and identifies research gaps.

Translation of research findings to policy statements—especially those concerning important public health conditions like HIV, and with the potential to be distorted and/or misunderstood by the media—can have implications for a breadth of stakeholders (Figure 3). In the case of POI use and HIV acquisition, policy changes would have significant impact for governmental HIV and family planning programs as we all as NGOs supporting these programs, particularly in countries with a high HIV burden. Finally, civil society and patient advocate groups provide the most direct feedback of policy impact from consumers of family planning and HIV services. Recognizing the potential for an unanticipated and unwarranted impact for these stakeholders, WHO strived to include all relevant participants at the most important plenary meetings (Figure 2).

In fact, we found engagement with stakeholders to be critical throughout this process. Discussions with these stakeholders ultimately led WHO to conclude that initial changes to the MEC from category 1 to category 1* were inadequate. While the initial intent was to retain a category 1 designation so that provision of POIs would not be adversely affected, concerns from stakeholders, particularly managers of national health programs, that the category 1* did not encourage adequate counseling led to a reevaluation of the data and revision of category 1* to category 2 in 2016. Similarly, once the WHO guidance was changed, we found direct engagement with stakeholders in the dissemination process facilitated transparency, increased buy-in to the changes, and encouraged collaborative strategizing with regards to implementation.

Engagement with stakeholders was critical throughout the process of considering WHO guideline changes.

From this experience, we acknowledge that the need for stronger communication and collaboration between the HIV and family planning communities is an area for improvement. While these communities have traditionally been siloed due to a variety of factors including the complex dynamics of donor funding, the organizational structure of ministry of health departments, and the providers of care in community health clinics, they share a large, important demographic of clients—women of reproductive age. In addition to integrating the messaging women receive so that the counseling around HIV and POI use can be optimized, integrating services could increase utilization of preventative services such as testing for HIV and other sexually transmitted infections (STIs) and ensure higher-quality reproductive health care for women living with HIV.^16^^,^^17^

### Messaging

The gradual, measured evolution of the change in guidance for POI use in women at high individual risk for HIV from MEC category 1 to category 2 reflects a very deliberate attempt to make recommendations that adequately addressed the analyses of observational data that had serious limitations while avoiding drastic, and perhaps unfounded, shifts in global family planning policy. However, communicating this change to patients is exceedingly challenging. Questions like, “What defines high risk?” and “When and how should this message be communicated with women?” have imperfect answers that are particular to local contexts. This also demonstrates the limitation of the use of policy documents in direct clinical care. A policy document that notes “a possible increased risk” seems reasonable in order to reflect the uncertainty of research. In real life, women come to health care encounters with specific goals (i.e., obtain injectable contraception), with preexisting notions about HIV risk from previous messaging or other information sources and with individual levels of fear with regards to HIV transmission and unintended pregnancy based on their life experiences. In the often-brief amount of time women have with providers at these encounters, a nuanced discussion about research uncertainty and values clarification may not be possible.

Communicating complex policy and research data to patients is challenging.

To address these challenges and to optimize the messaging of the most recent policy changes, WHO held an official dissemination meeting in Johannesburg, South Africa, in April 2017 with WHO, ministry of health representatives from 12 of the 14 African countries with HIV prevalence greater than 5%, health organization donors, researchers, representatives from affected populations, and advocacy groups. The meeting was conducted in 2 parts. During the first part, WHO reviewed the research with stakeholders so that understanding was harmonized. While many country and organizational leaders are aware of the general concerns around POIs and HIV acquisition, conveying the nuance would help clarify why the guidance is not straightforward.

The second part of the meeting was focused on implementation. In order to harmonize messaging, WHO did not provide specific messaging but rather a framework that was extensively discussed and edited so that it could reflect the major concerns and suggestions of the stakeholders and also address the issues they anticipated. Following, brief counseling messages were created, which include a series of tips for health care providers to discuss with clients. The dialogue suggests providing clients with 5 key facts about POIs and then 2 key questions to help clients consider whether they want to use POIs. The counseling tips will be included in the updated *Family Planning: A Global Handbook for Providers*, to be released at the end of 2017 (www.fphandbook.org). These meetings also gave WHO an opportunity to reinforce the most important recommendations that were not altered by the change in MEC category:

1. Women and girls at high risk for HIV should not be denied any method of contraception, and rights-based counseling is necessary for them to make an informed choice.
2. Policies and programs need to emphasize dual protection from unplanned pregnancy and STIs/HIV.
3. Women and girls should be given a range of contraceptive options from which they can choose for preventing unwanted pregnancy.

WHO will continue to assist health departments and service providers by providing education and suggested communication techniques through regional meetings, webinars, updated global references, counseling tools, and job aids.

Counseling messages and tips on POI use among women at risk for HIV will be included in the updated *Family Planning: A Global Handbook for Providers* (www.fphandbook.org).

### Research

Further research is needed to clarify the uncertainty surrounding POI use and HIV transmission. Most of the current research is observational and therefore limited by potential bias and confounding. Currently WHO, along with FHI 360, the University of Washington, and the Wits Reproductive Health and HIV Institute, are coordinating a large randomized controlled trial (RCT) (called Evidence for Contraceptive Options and HIV Outcomes [ECHO]) of almost 8,000 women across 12 clinical sites in 4 different countries to provide more definitive evidence about the association of HIV acquisition with use of 3 common contraceptive methods: the levonorgestrel-releasing implant (Jadelle), DMPA, and the copper-bearing IUD.^18^ The RCT is scheduled to be completed in 2018, and the findings are expected to provide important information that will inform future WHO guidance on this issue.

WHO and partners are coordinating a large RCT to provide more definitive evidence about the association of HIV acquisitions with use of Jadelle implants, DMPA injectables, and the copper IUD.

However, research should not be limited solely to the question of transmission. We also need a more rigorous study of attitudes and beliefs of women and health care workers in affected countries with regards to HIV and unintended pregnancy; while modeling may give us population-level projections of the impact of new policy, this does not necessarily reflect the values of individual women. Additionally, studies that help us understand not only the best avenues to reach women but also what communication strategies are most effective are sorely needed. This supporting body of research will ensure that the results of future scientific studies such as ECHO can be translated optimally into policy and practice.

### Monitoring and Evaluation

We cannot take for granted that the changes to guidelines will automatically result in improved messaging or safer provision of contraceptives. WHO continues to follow the implementation of new guidelines among the most affected countries. For example, through regional and country representatives, WHO regularly audits progress and problems from the countries most affected and provides technical assistance accordingly. Regional meetings—in person and by webinar—are held so that countries may share their experiences with each other and other stakeholders in a formal setting. For example, in early 2018, a virtual meeting among the representatives of the 12 countries attending the April Johannesburg meeting is planned with the purpose of following up on the progress of the national action plans that were developed during the meeting and defining plans for continued monitoring. These ongoing efforts will be ultimately reincorporated into future guidance.

## CONCLUSION

The process from publishing research to having impact on health outcomes is not straightforward. Often, major research findings rely on slow, organic infiltration into practice norms. Large normative health institutions such as WHO can shape, guide, and expedite this process by translating research into guidelines and systematically disseminating them to member states and organizations. However, the inputs into these guidelines—and the “translation” of guidelines into practice—are often imperfect as well. In the case of POI contraception and HIV acquisition, WHO sought to balance the risks of maternal morbidity and mortality associated with unintended pregnancy with the risk of HIV acquisition, ultimately putting forth recommendations that would serve to promote the “highest attainable standard of health” for women everywhere. However, depending on one's interpretation of the research, arguments can be made that the ultimate change from an MEC category 1 to category 2 was either premature or not timely enough. It remains to be seen, however, what the impact of these guidelines will be.

The translation of important, often-nuanced research findings from journal page to health policy to the provider-client interaction is complex and challenging work. This is made even more difficult as it is often the part of the process with the least funding and resources. WHO recognizes that even the best efforts are flawed and continues to learn from this experience on POIs and HIV acquisition. Future policy work can use these lessons to improve implementation, minimize harm, and continue disseminating important research to the global health community.

## Notes

**Peer Reviewed**
